# Optimization of ultrasound-assisted extraction of phenolics from *Asparagopsis taxiformis* with deep eutectic solvent and their characterization by ultra-high-performance liquid chromatography-mass spectrometry

**DOI:** 10.3389/fnut.2022.1036436

**Published:** 2022-11-17

**Authors:** Heqi Gao, Yuxi Wang, Zhiqiang Guo, Yuxin Liu, Qian Wu, Juan Xiao

**Affiliations:** ^1^State Key Laboratory of Marine Resource Utilization in South China Sea, Engineering Research Center of Utilization of Tropical Polysaccharide Resources, Ministry of Education/Key Laboratory of Food Nutrition and Functional Food of Hainan Province, School of Food Science and Engineering, Hainan University, Haikou, China; ^2^Key Laboratory of Fermentation Engineering, Ministry of Education, Hubei Key Laboratory of Industrial Microbiology, National “111” Center for Cellular Regulation and Molecular Pharmaceutics, Hubei Research Center of Food Fermentation Engineering and Technology, Hubei University of Technology, Wuhan, China

**Keywords:** phenolic, ultrasound-assisted extraction, *Asparagopsis taxiformis*, deep eutectic solvent, antioxidant ability

## Abstract

*Asparagopsis taxiformis* is a significant source of phenolics. Owing to the incessant demand of green extraction procedures for phenolics from *A. taxiformis*, ultrasound-assisted extraction (UAE) using deep eutectic solvents (DESs) was optimized. Among the tested DESs, betaine-levulinic acid afforded the highest total phenolic content (TPC). Moreover, the optimal extraction conditions elucidated from single-factor and response surface methodologies comprised a 52.41°C ultrasonic temperature, 46.48% water content of DES, and 26.99 ml/g liquid-to-solid ratio. The corresponding TPC (56.27 mg GAE/100 g DW) and antioxidant ability fitted the predicted values. UAE afforded superior TPC and antioxidant abilities with DESs than with traditional solvents. Using UHPLC-MS, seven phenolic acids, 18 flavonoids, and two bromophenols were identified and quantified. DES-UAE afforded the highest phenolic compound number (26) and sum of contents. These results disclose the high extraction efficiency of DES-UAE for *A. taxiformis* phenolics and provide a basis for the higher-value application of this species.

## Graphical Abstract

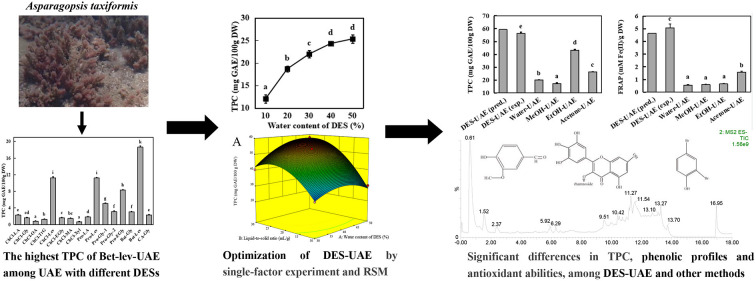

## Introduction

*Asparagopsis taxiformis*, a red alga belonging to the Bonnemaisoniaceae family, has been utilized in local food and traditional Chinese medicine for many centuries (1). Recent studies have revealed that *A. taxiformis* contains substantial quantities of protein, fat, fiber, and phytochemicals including polysaccharides, halogenated compounds, and phenolics (2, 3). Accumulating studies have focused on the antimethanogenic, antibacterial, and antifungal activities of halogenated compounds from *A. taxiformis* (3, 4). However, *A. taxiformis* is also rich in phenolics and other phytochemicals, which have not been well studied to date (2, 5). Owing to the health-promoting effect of phenolics extracted from terrestrial plants, there has been a growing interest in phenolics from *A. taxiformis*. In particular, a few studies have recently attributed the antimicrobial and antifungal activities of *A. taxiformis* extracts to the phenolics present in this species (2, 5). However, to the best of our knowledge, phenolic profiles of *A. taxiformis* have not been reported.

Marine algae are distinguished by particular phenolic classes that are not identified in terrestrial plants. In particular, bromophenols, which contain different numbers of benzene ring, hydroxyl, bromine, and other groups in their structure. They have shown beneficial biological activities including antidiabetic and anti-obesity activities and thus gained significant attention in the fields of pharmaceutical and food agents (6, 7). Bromophenols were first isolated from red seaweeds and subsequently found in green and brown seaweeds too (7). Notably, phenolics common to terrestrial plants, including flavonoids and phenolic acids, have also been found in numerous seaweeds (6).

Owing to their diversity, the high-efficiency extraction of phenolics from seaweeds is highly challenging. One major issue encountered in this process is the choice of a suitable extraction solvent. Organic solvents, particularly methanol, acetone, ethanol, and their aqueous mixtures, have been commonly utilized for the extraction of phenolic compounds from seaweeds (5, 8). However, no unanimous consensus on the best solvent for the extraction of phenolics from *A. taxiformis* has been reached. Mellouk et al. (5) reported that the highest TPC was obtained from methanolic and aqueous extracts of *A. taxiformis*, followed by those attained using ethanolic, hydroethanolic, and hydromethanolic extracts. On the other hand, Nunes et al. (2) showed that the highest TPC, antioxidant ability, and antiproliferative activity were obtained from methanolic extracts of *A. taxiformis*, followed by those from the chloroform, petroleum ether, and ethyl acetate extracts. Additionally, the safety of organic solvents has posed a challenge to the widespread use of organic solvents for extracting phytochemicals. Thus, extraction methods using green solvents are gaining significant attention.

Deep eutectic solvents (DESs), which constituted by hydrogen-bond acceptors and donors, are showing great potential as new green solvents for extracting phytochemicals (9, 10). In contrast with organic solvents, DESs are not only eco-friendly but also have the benefits of easy synthesis, high stability, low volatility, and wide polarity (10). Various studies have reported that compared with conventional solvents, DESs show superior extraction capacities for phenolics from terrestrial plants including *Corylus avellana, Morinda citrifolia*, and *Zingiber officinale* (10, 11). UAE has been demonstrated to enhance the extraction efficiency of phenolics, utilizing acoustic cavitation to destruct the cell-wall structure (12). Moreover, the use of DESs instead of traditional organic solvents in this process was reported to further increase the extraction efficiency of phenolics from *Moringa oleifera* and *Paederia scandens* (11, 13). However, research focusing on phenolic extraction from *A. taxiformis* using DES-UAE remains scarce.

With these facts in mind, in this study we first elucidated the most suitable DES for extracting phenolics from *A. taxiformis*. We then proceeded to optimize the DES-UAE process through single-factor experiment and subsequent response surface methodology, using the elucidated optimal DES as the solvent to maximize the TPC and antioxidant ability of *A. taxiformis*. Finally, we evaluated the differences in the TPC, phenolic profiles (qualitative and quantitative analysis; UHPLC-MS), and antioxidant abilities of the extracts gained from *A. taxiformis* by UAE using the optimal DES and traditional solvents.

## Materials and methods

### Materials and reagents

*Asparagopsis taxiformis* was collected from Wuzhizhou island, Sanya, Hainan Province, China (109°45.494′E, 18°18.555′N), in May 2020, and characterized by Prof. Xiubao Li from Hainan University. The seaweed was rinsed in water to eliminate any visible surface contaminants, freeze-dried, and subsequently crushed through a 60-mesh sieve using a food grinder (Tianjin Taist Instrument Co., Ltd., Tianjin, China). It was then stored at −18°C until further use.

For the UHPLC-MS analysis, 4-bromophenol and 2,4-dibromophenol were purchased from Tanmo Quality Inspection Technology Co., Ltd. (Beijing, China). Quercitrin, apigenin, hesperidin, diosmetin, and acacetin were acquired from Qiyun Biotechnology (Guangzhou, China), leucocyanidin from Shanghai Pureone Biotechnology Co., Ltd. (Shanghai, China), aromadendrin from Shanghai Tauto Biotech Co., Ltd. (Shanghai, China), and all other phenolic standards from Sigma were acquired. The DES-related reagents and total antioxidant capacity assay kits were, respectively acquired from Aladdin Biochemical Technology Co., Ltd. (Shanghai, China) and Nanjing Jiancheng Bioengineering Institute (Nanjing, China).

### Preparation and physicochemical properties of the DESs

Sixteen DESs comprising choline chloride, L-proline, betaine, and citric acid as hydrogen-bond acceptors and different hydrogen-bond donors (polyalcohols and organic acids) were prepared according to previously reported methods (9). Briefly, the hydrogen-bond acceptors and donors (Table 1) were mixed at a specific molar ratio, as shown in Table 1. After a certain ratio of water (w/w) was added, the mixture was continuously stirred on a magnetic stirring apparatus (Eyelan-1100, Tokyo Physicochemical Instrument Co., Ltd., Tokyo, Japan), at 80°C, to form the transparent and homogeneous DESs. Subsequently, the density, polarity and viscosity of DESs were determined according to previously reported methods (14, 15). The density was determined by weighting 1 cm^3^ of DES at analytical balances at room temperature (14). The polarity was measured with Nile red as an indicator by scanning at the spectral range of 400–800 nm in a UV-Vis spectrometer and presented using the molar transition energy (*E*_NR_), calculated using the formula: *E*_NR_ (kcal/mol) = 28,591/λ_max_ (14). The viscosity was measured using TA Instruments Discovery HR-2 rheometer with the shear rate of 0.1/s at atmospheric pressure (15).

**Table 1:** DESs and their physicochemical properties.

**No**.	**Solvent abbreviation**	**Hydrogen bond acceptor**	**Hydrogen bond donor**	**Molar ratio**	**Density (g/cm^3)^**	**Polarity (kcal/mol)**	**Viscosity (mPa·s)**
1	ChCl-LA	Choline Chloride	Lactic acid	1:3	1.16 ± 0.005	47.89 ± 0.09	211.70 ± 3.35
2	ChCl-Gly	Choline Chloride	Glycerin	1:2	1.17 ± 0.004	48.79 ± 0.08	323.00 ± 4.29
3	ChCl-OA	Choline Chloride	Oxalic acid	1:1	1.19 ± 0.004	44.67 ± 0.13	2,533.50 ± 18.84
4	ChCl-TrG	Choline Chloride	Triethylene glycol	1:4	1.12 ± 0.002	49.81 ± 0.08	195.50 ± 1.83
5	ChCl-Lev	Choline Chloride	Levulinic acid	1:2	1.12 ± 0.001	49.13 ± 0.15	25.80 ± 0.74
6	ChCl-EGly	Choline Chloride	Ethylene glycol	1:2	1.11 ± 0.002	49.21 ± 0.14	266.10 ± 3.58
7	ChCl-MA	Choline Chloride	Malic acid	1:1	1.22 ± 0.004	47.89 ± 0.11	407.80 ± 5.81
8	ChCl-Xyl	Choline Chloride	Xylitol	1:1	1.20 ± 0.001	48.79 ± 0.11	566.70 ± 1.61
9	Pro-LA	l-Proline	Lactic acid	1:2	1.21 ± 0.002	49.64 ± 0.09	128.60 ± 1.17
10	Pro-Lev	l-Proline	Levulinic acid	1:2	1.16 ± 0.001	49.98 ± 0.14	84.70 ± 1.48
11	Pro-Gly-1	l-Proline	Glycerin	1:2	1.22 ± 0.004	49.21 ± 0.10	236.40 ± 0.87
12	Pro-Gly-2	l-Proline	Glycerin	1:2.5	1.22 ± 0.001	49.13 ± 0.07	379.50 ± 6.25
13	Pro-EGly	l-Proline	Ethylene glycol	1:2	1.16 ± 0.001	57.18 ± 0.12	172.00 ± 4.55
14	Bet-Gly	Betaine	Glycerin	1:1	1.17 ± 0.003	49.55 ± 0.11	523.90 ± 5.29
15	Bet-Lev	Betaine	Levulinic acid	1:2	1.13 ± 0.005	49.98 ± 0.10	300.10 ± 6.04
16	CA-Gly	Citric acid	Glycerin	1:2	1.29 ± 0.005	47.49 ± 0.13	827.00 ± 8.40

All DESs contained 20% of water (w/w).

### Extraction of phenolics

#### Screening of DESs

*Asparagopsis taxiformis* powder (1 g) was placed in glass tubes and blended with 10 ml DES containing 20% water (w/w) in triplicate. Subsequently, extraction was performed using an SB25-12DTD ultrasonic machine (40 kHz, Ningbo Scientz Biotechnology Co., Ltd., Ningbo, China) at 320 W, for 10 min, at 30°C. The mixtures were centrifugated (10,000 rpm) for 15 min and then TPC of the supernatants was measured to evaluate the extraction efficiency.

#### Single-factor experiment

The single-factor experiment aimed to assess the effects of five factors—water content of DES (10 20, 30, 40, and 50%); liquid-to-solid ratio (10, 20, 30, and 40 ml/g); ultrasonic temperature (30, 40, 50, and 60°C); ultrasonic time (0, 5, 10, 20, 30, and 40 min); and ultrasonic power (240, 280, 320, 360, 400, 440, and 480 W)—on the TPC of the *A. taxiformis* extracts. The other parameters were kept constant: water content of DES (20%), ultrasonic temperature (30°C), liquid-to-solid ratio (20 ml/g), ultrasonic time (10 min) or ultrasonic power (320 W).

#### Response surface methodology

According to the single-factor experiment, the major influential variables—water content of DES (A, 30, 40, and 50%); liquid-to-solid ratio (B, 10, 20, and 30 ml/g); and ultrasonic temperature (C, 40, 50, and 60°C)—were applied to a Box-Behnken design using Design-Expert v. 8.0.5 to assess their influence on TPC, ABTS value, and FRAP value at a constant ultrasonic time (10 min) and ultrasonic power (360 W).

#### Verification and comparison experiments

*Asparagopsis taxiformis* powder (1 g) was mixed with 27.0 ml Bet-Lev (46.5% water; DES-UAE), water (Water-UAE), 70% methanol (MeOH-UAE), 70% ethanol (EtOH-UAE), or 70% acetone (Acetone-UAE). After ultrasound extraction (360 W) at 52.0°C for 10 min, the mixtures were centrifugated (10,000 rpm) for 15 min to acquire the supernatants.

### Measurement of the TPC

After determination using the Folin-Ciocalteu colorimetric method (8), TPC was quantified using the standard curve of gallic acid (mg GAE/100 g DW).

### Phenolic characterization and quantification using UHPLC-MS

The phenolic compounds were monitored using UHPLC-MS (Xevo TQ-S Micro, Waters, Milford, USA) according to our previously reported method with slight modifications (13). The Acquity UHPLC BEH-C18 column was eluted by water with 0.25% formic acid (A) and methanol with 0.25% formic acid (B) at a gradient elution program (0–1 min, 95% A; 8 min, 75% A; 11 min, 40% A; 13–16 min, 0% A; and 16.2–18 min, 95% A). The basic compound structures were then tentatively deduced according to the characteristic ions characterized using multiple reaction monitoring as well as the published mass spectral data (7, 16–27). Subsequently, the retention times and mass spectral data of the phenolic standards were compared to those of the aforementioned tentative basic structures to finally assign the phenolics. The contents were calculated using the standard curve of each standard and expressed in μg/g DW. The mass spectra were recorded using the following parameters: mass spectral range of 50–1,000 *m*/*z*, cone voltage of 30 V, capillary voltage of 2.0 kV, drying gas flow of 1,000 L/h, and drying gas (N_2_) temperature of 500°C.

### Antioxidant activity

To determine the antioxidant activity, ABTS and FRAP assay kits were employed following the manufacturer's protocols, using Trolox and FeSO_4_ as the standards, respectively (13). Their calibration curves were then utilized to calculate the ABTS and FRAP values, which were respectively expressed in mM TE/g DW and mM Fe(II)E/g DW.

### Statistical analyses

All data are shown as the mean values ± standard deviation (*n* = 3). Statistical analyses were conducted through one-way ANOVA and subsequent Duncan's *post hoc* test at *p* < 0.05 level using SPSS 16.0 software.

## Results and discussion

### Effects of the DESs on the TPC

**Figure 1:**
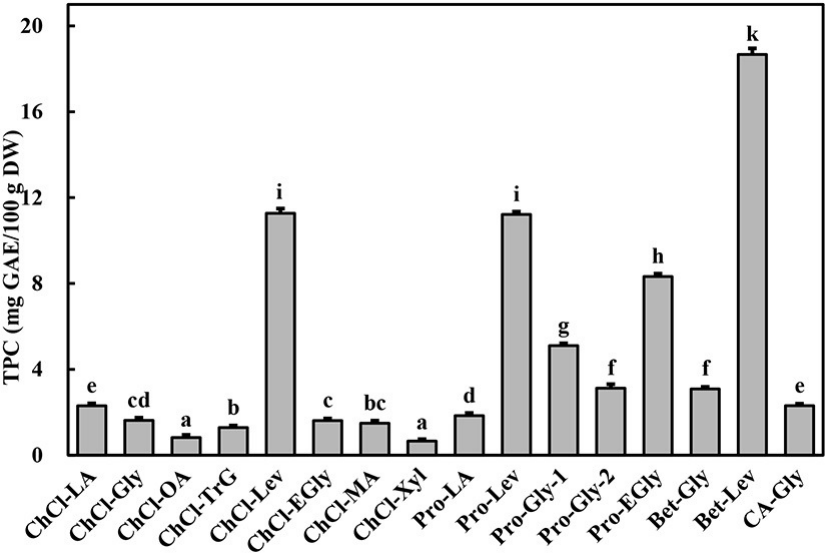
Total phenolic content (TPC) of *Asparagopsis taxiformis* extracts gained by UAE with different DESs. Different letters indicate significant differences (*p* < 0.05).

The extraction effectiveness is attributed to the interaction between the solubilization ability of the solvent and relative solubility of the phenolic in the solvent, which resolves the distribution coefficient and extractability (28). DESs have shown excellent extractability for phytochemicals, including phenolic compounds, in terrestrial plants (9, 10). This extractability is mainly associated with the polarity and viscosity of the DES, which in turn depend on its constituents and their molar ratios (9). Alcohols, amines, organic acids, and amino acids have been commonly used to prepare DESs (10). In particular, DESs comprising choline chloride, l-proline, betaine, and citric acid as hydrogen-bond acceptors and polyalcohols and organic acids as hydrogen-bond donors show high extractability for phenolics (9, 11). Importantly, water is usually added to the DES to increase its polarity and reduce its viscosity (11, 13). In this study, marked differences were found among the TPC of *A. taxiformis* extracts obtained by UAE with various DESs (*p* < 0.05, Figure 1). Specifically, Bet-Lev afforded the highest TPC (18.67 ± 0.29 mg GAE/100 g DW), followed by Pro-Lev and ChCl-Lev, which afforded values that were 60.07 and 60.36% that of the TPC extracted by Bet-Lev (*p* < 0.05). ChCl-Xyl and ChCl-OA afforded the lowest TPC, which were 3.51 and 4.42% that of the TPC extracted by Bet-Lev (*p* < 0.05). Many studies have reported that different DESs show remarkably distinct extractabilities for phenolics (11, 13). Indeed, Bet-Lev (1:2) was more efficient than ChCl-Lev, Pro-Lev, ChCl-Xyl, and ChCl-OA in extracting phenolics from *M. oleifera* but far less efficient than Pro-Gly-2 (1:2.5) (11). Moreover, Bet-Lev (1:2) presented higher extraction yields for phenolics from strawberry and raspberry waste than those afforded by ChCl-Gly (1:2) and another four DESs (29). Liu et al. (13) showed that Bet-Lev (1:2) was less efficient than ChCl-TrG (1:4). However, it displayed a similar efficiency to that of ChCl-Xyl in the extraction of phenolics from *P. scandens* (13). In addition, when different DESs were used, significantly distinct phenolic profiles were obtained from strawberry and raspberry waste (29). The similarity in the polarity of the DES and phenolic compounds is a key factor of the extraction efficiency (9, 13). Moreover, a low DES viscosity results in high mass transfer and compound diffusion (10). Thus, in this study we supposed that the highest TPC was obtained with Bet-Lev because of the adequate viscosity of this DES together with its highly similar polarity to that of the phenolics in *A. taxiformis*. Therefore, we selected Bet-Lev as the DES for the subsequent experiments.

### Single-factor experiment of DES-UAE

The properties of a phytochemical extraction method, including the extraction solvent, parameters, and type of assisted technology, all contribute to the extraction efficiency (28, 30). In addition, the water content significantly affects the DES polarity and viscosity, which in turn significantly affect the extraction efficiency (10). Moreover, both the ultrasonic conditions (power, time, and temperature) and liquid-to-solid ratio were reported to greatly affect the extraction efficiency (11, 13).

**Figure 2:**
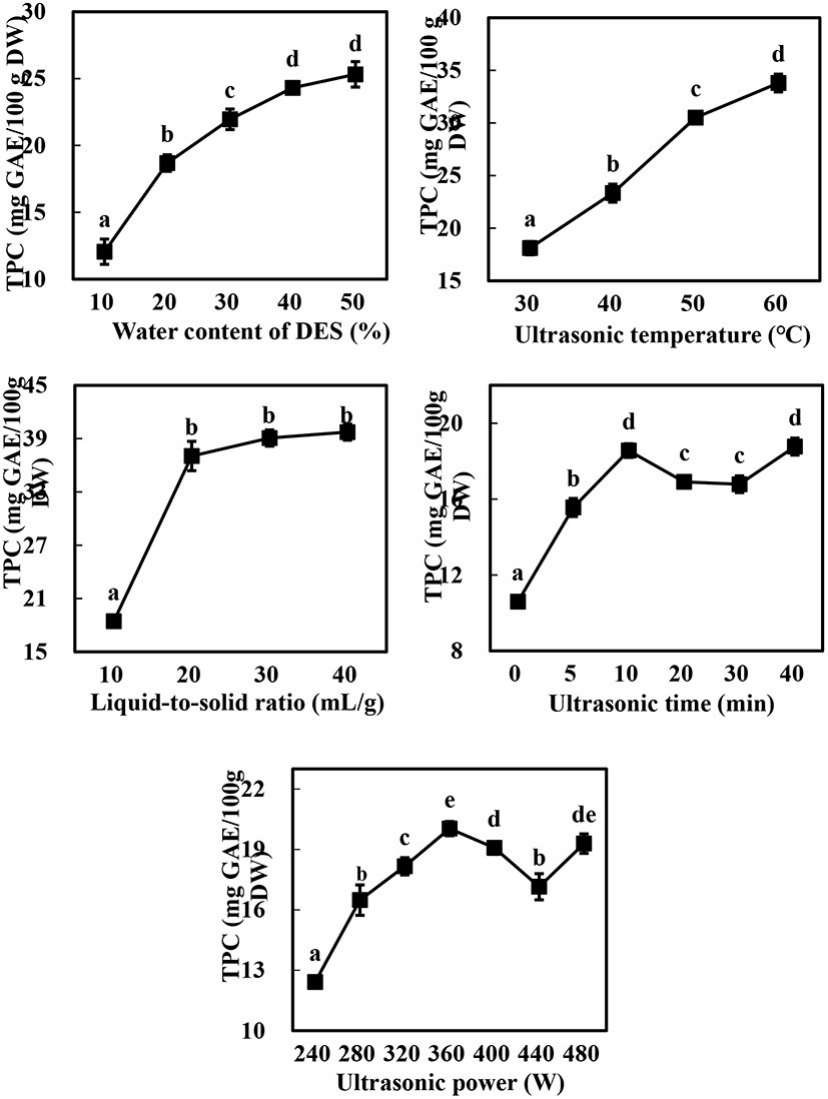
The effect of water content of DES, liquid-to-solid ratio, ultrasonic temperature, ultrasonic time and ultrasonic power on the TPC of *Asparagopsis taxiformis* extracts. Different letters indicate significant differences (*p* < 0.05).

As expected (10, 13), there were marked differences in the TPC of the extracts obtained using Bet-Lev with different water contents (10, 20, 30, 40, and 50%) under identical UAE parameters (*p* < 0.05, Figure 2). With the growing water content of DES, TPC first raised markedly and subsequently more slowly until it reached the highest value at 50% water content of DES. Specifically, compared with the TPC extracted by Bet-Lev with 10% water content, the TPC extracted by Bet-Lev with 20, 30, 40, and 50% water contents increased 1.55-, 1.82-, 2.02-, and 2.10-fold, respectively (*p* < 0.05). Insignificant difference was found between the TPC extracted by Bet-Lev with 40 and 50% water contents (*p* > 0.05). Compared to that at the 10 ml/g liquid-to-solid ratio, the TPC increased by a significant 100.82% at 20 ml/g (*p* < 0.05). Conversely, there was no marked difference in the TPC extracted by Bet-Lev at 20, 30, and 40 ml/g liquid-to-solid ratios (*p* > 0.05). The increasing ultrasonic temperature also led to a significant increase in the TPC (*p* < 0.05), which reached a maximum at an ultrasonic temperature of 60°C. Notably, the TPC obtained at 60°C was 1.87-fold higher than that obtained at 30°C (*p* < 0.05).

The TPC increased from 10.06 ± 0.29 to 18.56 ± 0.36 mg GAE/100 g DW as the ultrasonic time was increased from 0 to 10 min, significantly decreased as the ultrasonic time was further increased to 20 and 30 min, and subsequently increased markedly to the level observed at 10 min (*p* < 0.05). Similarly, the TPC raised when the ultrasonic power was grew from 240 to 360 W, followed by a decrease as ultrasonic power further grew to 440 W and then an increase again as ultrasonic power finally grew to 480 W. These results were linked to acoustic implosion cavitation, which disrupts the structure of the plant cell wall, thereby promoting the dissolution of the phytochemicals present in the vacuoles and releasing the phytochemicals bound to the cell wall components (12, 31). Indeed, the increase in ultrasonic time and power has been widely reported to increase the extraction yield of phenolics (13, 28); however, the phenolics may be degraded if the ultrasonic power is too high or the ultrasonic time too low (12). The increase in the TPC at 40 min or < 480 W may be therefore attributed to the release of the bound phenolics from the cell wall components and their subsequent distribution in the solvent (31). Thus, water contents of DES (A) of 30, 40, and 50%; liquid-to-solid ratios (B) of 10, 20, and 30%; and ultrasonic temperatures (C) of 40, 50, and 60 °C were selected for the subsequent experiments at a constant ultrasonic time (10 min) and ultrasonic power (360 W).

### Modeling and optimization of DES-UAE

#### Model fitting

The TPC, FRAP, and ABTS values varied within the ranges of 24.26–57.82 mg GAE/100 g DW, 1.30–4.60 mM Fe(II)/g DW, and 1.67–4.78 mM TE/g DW, respectively (Table 2). Moreover, a highly significant model (*p* < 0.001, Table 3) and insignificant loss of fit (*p* = 0.0995, 0.8433, and 0.6377 for TPC, ABTS and FRAP, respectively) were observed. Good model accuracy as well as a high correlation was also observed, as indicated by the high *R*^2^ (0.9915, 0.9668, and 0.9676 for TPC, ABTS, and FRAP, respectively) and adjusted *R*^2^ values (0.9763, 0.9070, and 0.9091 for TPC, ABTS, and FRAP, respectively) and verified by the low coefficient of variation.

**Table 2:** Box-Behnken design and experimental responses.

**No**.	**A: water content of DES (%)**	**B: liquid-to-solid ratio (ml/g)**	**C: ultrasonic temperature (°C)**	**TPC** **(mg GAE/100 g DW)**		**FRAP** **(mM Fe(II)/g DW)**		**ABTS** **(mM TE/g DW)**	
				**Exp**.	**Pred**.	**Exp**.	**Pred**.	**Exp**.	**Pred**.
1	40 (0)	20 (0)	50 (0)	51.98 ± 0.26	52.67	3.97 ± 0.13	3.94	4.78 ± 0.24	4.30
2	50 (+1)	20 (0)	60 (+1)	53.74 ± 1.57	54.94	3.96 ± 0.22	4.09	3.74 ± 0.12	3.81
3	30 (−1)	20 (0)	60 (+1)	40.86 ± 0.25	39.85	3.17 ± 0.13	3.09	2.69 ± 0.12	2.60
4	40 (0)	20 (0)	50 (0)	52.67 ± 0.32	52.67	4.25 ± 0.13	3.94	4.06 ± 0.11	4.30
5	40 (0)	10 (−1)	40 (−1)	24.26 ± 1.56	25.02	1.30 ± 0.11	1.45	1.67 ± 0.12	1.56
6	40 (0)	20 (0)	50 (0)	53.37 ± 0.15	52.67	3.61 ± 0.13	3.94	4.06 ± 0.11	4.30
7	50 (+1)	30 (+1)	50 (0)	57.82 ± 0.38	57.38	4.60 ± 0.12	4.62	4.78 ± 0.22	4.59
8	30 (−1)	20 (0)	40 (−1)	40.97 ± 0.67	39.77	2.37 ± 0.12	2.24	2.63 ± 0.12	2.56
9	40 (0)	10 (−1)	60 (+1)	32.56 ± 0.29	33.12	2.04 ± 0.13	2.13	2.40 ± 0.16	2.30
10	50 (+1)	10 (−1)	50 (0)	29.87 ± 0.21	28.10	2.71 ± 0.12	2.48	1.68 ± 0.13	1.70
11	30 (−1)	30 (+1)	50 (0)	40.42 ± 0.39	42.19	3.02 ± 0.23	3.25	2.64 ± 0.14	2.62
12	40 (0)	30 (+1)	40 (−1)	47.94 ± 0.33	47.38	3.05 ± 0.16	2.96	3.34 ± 0.11	3.44
13	30 (−1)	10 (−1)	50 (0)	27.50 ± 0.47	27.94	1.96 ± 0.23	1.94	2.02 ± 0.13	2.21
14	40 (0)	30 (+1)	60 (+1)	55.04 ± 0.58	54.28	4.22 ± 0.12	4.07	3.61 ± 0.12	3.72
15	50 (+1)	20 (0)	40 (−1)	39.02 ± 0.78	40.03	3.08 ± 0.12	3.16	2.73 ± 0.12	2.82

Exp, experimental value; Pred, predicted value.

**Table 3:** ANOVA for response surface quadratic model.

**Term**	**Df**	**Sum of square**		
		**TPC**	**ABTS**	**FRAP**
Model	9	1,675.14^***^	14.18^**^	12.48^**^
A	1	117.81^**^	1.09^*^	1.83^**^
B	1	946.78^***^	5.45^**^	5.92^**^
C	1	112.58^**^	0.54^ns^	1.61^**^
AB	1	56.48^**^	1.54^*^	0.17ns
AC	1	54.98^**^	0.23^ns^	0.001612^ns^
BC	1	0.36^ns^	0.053^ns^	0.046^ns^
A^2^	1	93.67^**^	1.63^**^	0.13^ns^
B^2^	1	281.67^***^	2.71^**^	1.72^**^
C^2^	1	58.76^**^	1.75^**^	1.37^**^
Residual	5	14.31	0.49	0.42
*R* ^2^		0.9915	0.9668	0.9676
Adjusted *R*^2^		0.9763	0.9070	0.9091
Predicted *R*^2^		0.8723	0.7924	0.7002
Coefficient of variation		3.92	10.00	9.17
Model (*F*-value)		65.03	16.16	16.36
Model (*p*-value)		< 0.0001	0.0034	0.0033
Lack of fit (*F*-value)		9.21	0.27	0.69
Lack of fit (*p*-value)		0.0995	0.8433	0.6377

* Significant at p < 0.05.

** Highly significant at p < 0.01.

*** Extremely significant at p < 0.0001.

ns, not significant (p > 0.05).

#### Effects of the variables on the TPC

Apart from the linear terms (A, B, C), TPC was also remarkably influenced by the secondary (A^2^, B^2^, C^2^) and interactive (AB, AC) terms (Table 3). Among them, the liquid-to-solid ratio (B) and B^2^ exhibited a highly significant effect on the TPC (*p* < 0.0001). Numerous studies have reported that the liquid-to-solid ratio is one of the main factors that affects the extraction yield of phytochemicals including phenolics (9, 13). An increase in the liquid-to-solid ratio led to more than a twofold increase in the extraction yield (9). Equation 1 shows the relationship between the TPC and experimental variables:


(1) $$\begin{array}{l} {\text{Y}}_{\text{TPC}}=52.67+3.84\text{A}+10.88\text{B}+3.75\text{C}+3.76\text{AB}\\ \;+3.71\text{AC }0.30\text{BC }5.04{\text{A}}^{2}\;8.73{\text{B}}^{2}\;3.99{\text{C}}^{2} \end{array}$$

**Figure 3:**
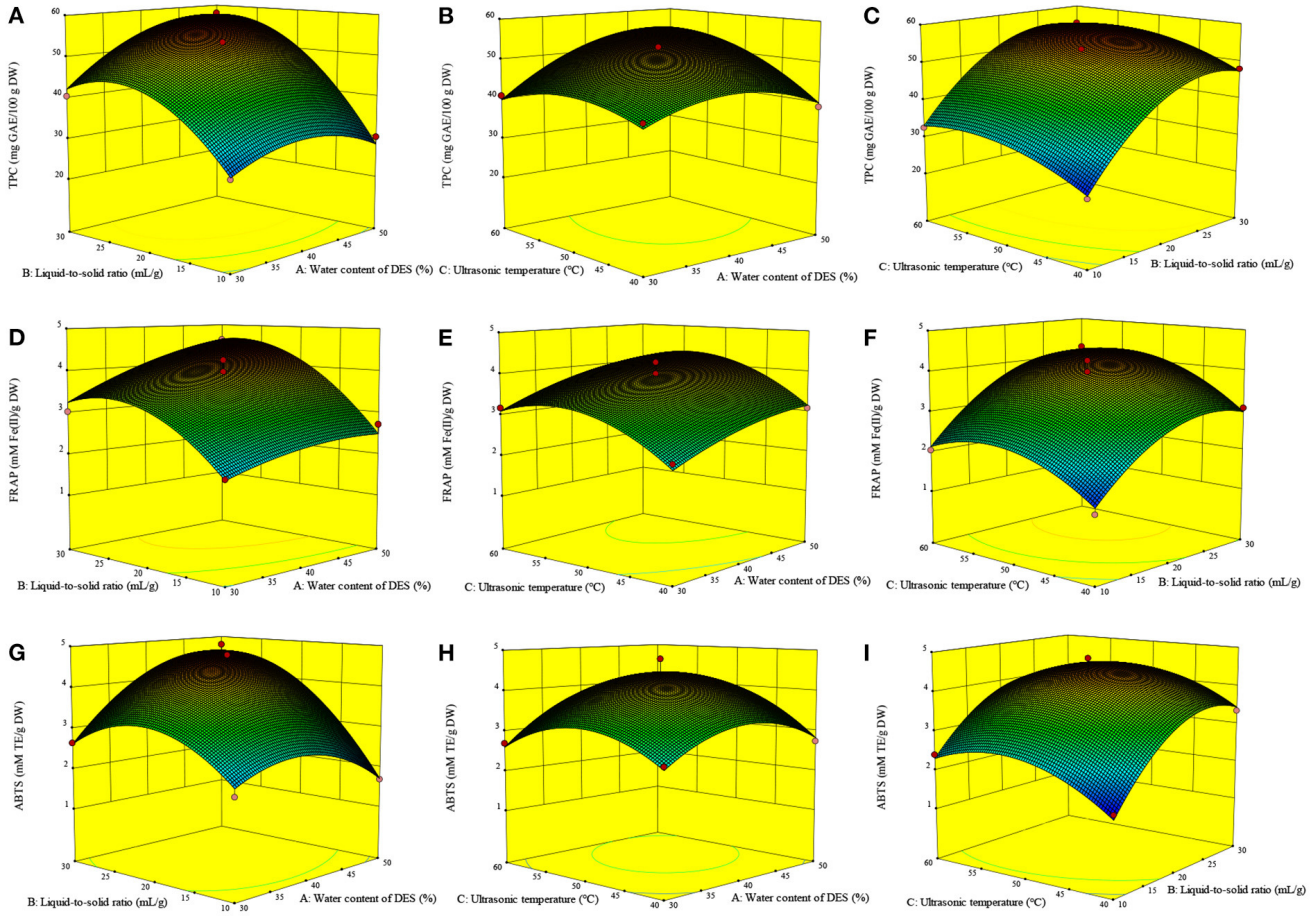
Interactive effects of the variables on the TPC **(A–C)**, FRAP **(D–F)** and ABTS **(G–I)** of *Asparagopsis taxiformis* extracts. Interactive effects of water content of DES and liquid-to-solid ratio on TPC **(A)**; Interactive effects of water content of DES and ultrasonic temperature on TPC **(B)**; Interactive effects of liquid-to-solid ratio and ultrasonic temperature on TPC **(C)**; Interactive effects of water content of DES and liquid-to-solid ratio on FRAP **(D)**; Interactive effects of water content of DES and ultrasonic temperature on FRAP **(E)**; Interactive effects of liquid-to-solid ratio and ultrasonic temperature on FRAP **(F)**; Interactive effects of water content of DES and liquid-to-solid ratio on ABTS **(G)**; Interactive effects of water content of DES and ultrasonic temperature on ABTS **(H)**; Interactive effects of liquid-to-solid ratio and ultrasonic temperature on ABTS **(I)**.

The three-dimensional response surfaces plots (Figures 3A–C) show the interactive effect of the experimental variables on the TPC. Figure 3A illustrates the marked interactive effect of water content of DES (A) and liquid-to-solid ratio (B) on the TPC. Specifically, the TPC increased sharply with the growth of these variables and reached the highest value at ~46% (A) and 27 ml/g (B), after which it decreased slightly. It was recognized that a high liquid-to-solid ratio facilitates compound diffusion (11). The increasing water content of DES leads to an increase in the DES polarity, resulting in its high extractability for phenolics with relatively high polarity over those with weak polarity (9, 10). An increased water content of DES also contributes to a reduction in the DES viscosity, resulting in an increase in mass transfer and compound diffusion (10). The slight decrease observed may therefore be due to the strong influence of water content of DES on the TPC. The significant interactive effect of water content of DES (A) and ultrasonic temperature (C) on the TPC is illustrated in Figure 3B. The TPC gradually increases with the increase of these two variables, with the highest TPC value observed at ~27 ml/g and 52°C.

#### Effects of the variables on the antioxidant ability

The FRAP value was markedly influenced by A, B, C, B^2^, and C^2^ (*p* < 0.01, Table 3). Meanwhile, the ABTS value was markedly influenced by B, A^2^, B^2^, and C^2^ (*p* < 0.01) and, to a lesser extent, AB and A (*p* < 0.05). Figure 3G shows the significant interactive effect between water content of DES (A) and liquid-to-solid ratio (B) on the ABTS value, which first sharply increased with the increase in these variables and then declined. Equations 2, 3 show the relationship between the antioxidant ability (FRAP and ABTS, respectively) and variables. Three-dimensional surface plots for FRAP (Figures 3D–F) and ABTS (Figures 3G–I) were constructed on the basis of these equations.


(2) $$\begin{array}{l} {\text{Y}}_{\text{FRAP}}=3.94+0.48\text{A }+\;0.86\text{B }+\;0.45\text{C }+\;0.21\text{AB}\\ \;+\;0.020\text{AC }+\;0.11\text{BC }0.19{\text{A}}^{2}\;0.68{\text{B}}^{2}\;0.61{\text{C}}^{2} \end{array}$$

(3) $$\begin{array}{l} {\text{Y}}_{\text{ABTS}}\;=\;4.30\;+\;0.37\text{A }+\;0.83\text{B }+\;0.26\text{C }+\;0.62\text{AB}\\ \;+\;0.24\text{AC }0.12\text{BC }0.66{\text{A}}^{2}\;0.86{\text{B}}^{2}\;0.69{\text{C}}^{2} \end{array}$$

### Verification experiment based on the optimal extraction parameters

**Figure 4:**
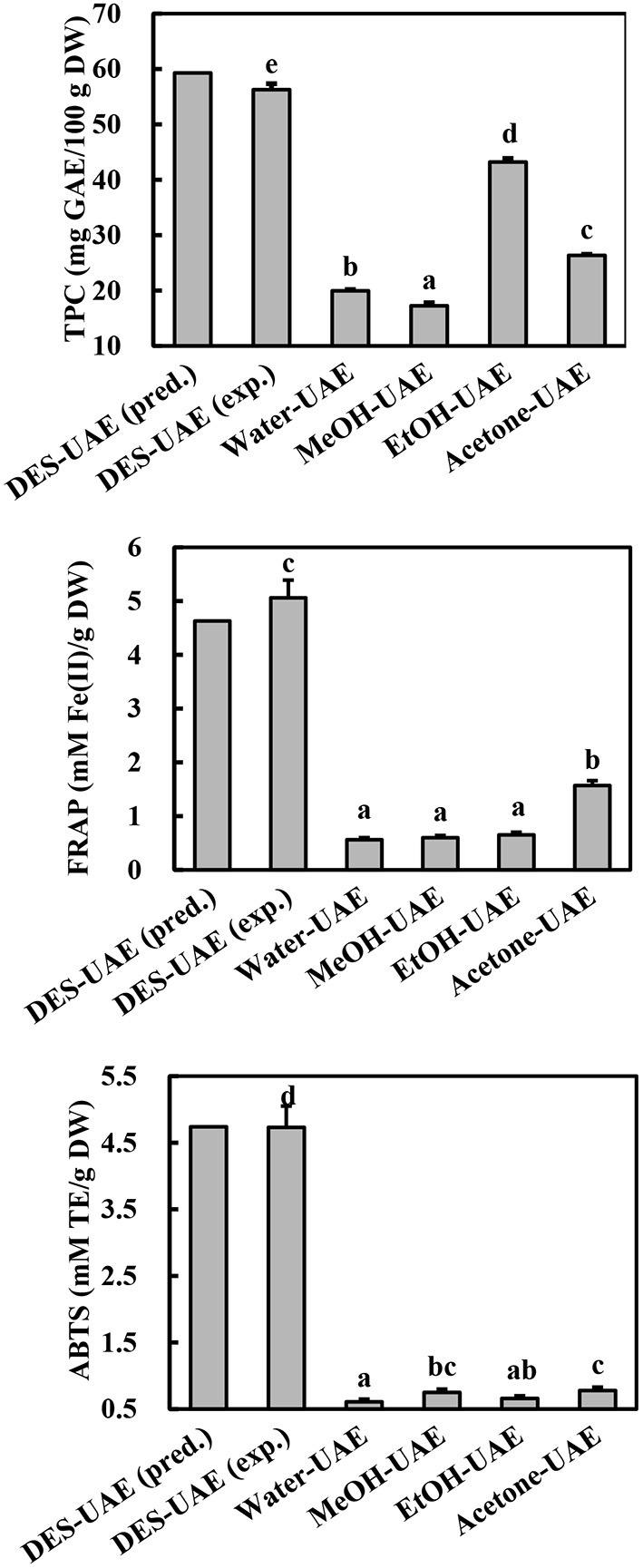
TPC and antioxidant ability of *Asparagopsis taxiformis* extracts gained by DES-UAE and UAE with traditional solvents. Different letters indicated significant differences (*p* < 0.05). DES-UAE, Water-UAE, MeOH-UAE, EtOH-UAE, Acetone-UAE: ultrasound-assisted extraction with deep eutectic solvent (Bet-Lev), water, 70% methanol, 70% ethanol and 70% acetone, respectively. exp.: experimental value; pred.: predicted value.

Based on the regression analysis achieved from the Box-Behnken design, we determined that the optimal conditions for extracting phenolics from *A. taxiformis* are water content of DES of 46.48%, ultrasonic temperature of 52.41°C, and liquid-to-solid ratio of 26.99 ml/g. To investigate the rationality of the Box-Behnken design, the verification experiment was conducted under water content of DES of 46.50%, ultrasonic temperature of 52.00°C, and liquid-to-solid ratio of 27.00 ml/g. As shown in Figure 4, the experimental values for TPC, FRAP, and ABTS (56.27 ± 1.08 mg GAE/100 g DW, 5.06 ± 0.33 mM Fe(II)/g DW, and 4.73 ± 0.32 mM TE/g DW, respectively) approached the predicted values, revealing the rationality of the Box-Behnken design.

### Comparison of DES and traditional solvent extractants in UAE

#### TPC and antioxidant ability

UAE methods using traditional solvents (organic aqueous mixtures and water) have been commonly applied to extract phenolics from marine algae (30). To confirm the high efficiency of optimized DES-UAE process, traditional solvents (water, 70% methanol, 70% ethanol, and 70% acetone) were also utilized in the UAE to extract phenolics from *A. taxiformis*. From the comparative study, DES-UAE presented the highest TPC, FRAP, and ABTS values (Figure 4, *p* < 0.05), which were respectively 1.30–3.27-, 3.22–9.03-, and 6.03–7.79-fold greater than those extracted using the other listed methods. These results indicate the high efficiency of the DES-UAE process for extracting phenolics from *A. taxiformis*. This well agrees with the literature, which reports that DES-UAE is a highly efficient method to extract phenolics (10, 11, 13). For example, for extracting phenolics from *M. oleifera*, DES-UAE afforded remarkably higher total flavonoid content and antioxidant abilities than UAE using traditional solvents (11). Similar trends were also observed in the extraction of phenolics from *Curcuma longa* and *P. scandens* (9, 13).

#### Characterization of phenolics using UHPLC-MS

As shown in Table 4, seven phenolic acids (peaks 1–7), 18 flavonoids (peaks 8–25), and two bromophenols (peaks 26–27) were identified from *A. taxiformis* by UHPLC-MS, along with many unknown compounds. The precursor ion (*m*/*z* 152.99) of peak 1 generated fragment ions at *m*/*z* 138.0 [M + H–CH_3_]^+^, 125.0 [M + H–CO]^+^, and 93.0 [M + H–CO–CH_3_-OH]^+^, and thus, peak 1 was assigned as vanillin (16). Ethyl vanillin (peak 2), with its parent ion at *m*/*z* 164.95, was identified through the product ions at *m*/*z* 136.2 [vanillin–H–CH_3_]^−^ and 92.05 [vanillin–H–CH_3_-CO_2_]^−^ (17). Peaks 3–5, with precursor ions at *m*/*z* 137.0, 163.1, and 193.0, were identified as 2-hydroxybenzoic, *p*-coumaric, and ferulic acids through the loss of CO_2_, CH_3_, or/and C_2_H_4_ group/s, respectively (18, 19). Rosmarinic acid (*m*/*z* 358.96) generated fragment ions at *m*/*z* 196.96 and 161.0, which were assigned as the dihydroxyphenyl-lactic acid moiety and caffeic acid with a loss of one molecule of water, respectively (20). *Trans*-cinnamic acid (*m*/*z* 146.95) generated a fragment ion at *m*/*z* 118.94 through CO loss (18). With the same parent ions, catechin (peak 8) and epicatechin (peak 9) presented identical product ions [M–H−44]^−^ (CO_2_ loss) and [M–H−152]^−^ (RDA fragmentation), respectively, but with distinct retention times (18, 21). Peaks 10–12 were assigned as epigallocatechin, gallocatechin, and epigallocatechin gallate according to their parent ions at *m*/*z* 304.98, 304.98, and 456.9, respectively, and as products ions based on C_6_H_6_O_3_ loss or RDA fragmentation ([M–H−152]^−^) together with their retention times (21). Peak 13 was identified as quercetin based on its parent ion (*m*/*z* 301.0) and representative fragment ions derived from RDA fragmentation (*m*/*z* 179.0 and 151.0) (18–20). Peaks 14–18 were identified as myricitrin, quercitrin, quercetin-3-O-α-l-arabinoside, kaempferol-3-O-rutinoside, and hesperidin, respectively, through cleavage of the rhamnoside, arabinoside, or rutinoside group (11, 19, 22). Peaks 19 (acacetin) and 22 (diosmetin) displayed parent ions at *m*/*z* 301.0 and 285.0, respectively, with an identical fragment ion (*m*/*z* 153.0) resulting from RDA fragmentation (23, 24). With identical parent ions (*m*/*z* 271.0), peaks 20 and 21 were, respectively authenticated as baicalein and apigenin from their fragment ions, which corresponded to those previously reported in the literature (18, 22). Peak 23 (*m*/*z* 286.8) was assigned as aromadendrin through a fragment ion (*m*/*z* 258.97) resulting from CO loss, which was previously reported by Venditti et al. (25). Peak 24 (*m*/*z* 306.92) and peak 25 (*m*/*z* 286.91) produced fragments ions at *m*/*z* 155.1 [M–H−152]^−^ and 126.99 [M–H−152–CO]^−^ for leucocyanidin and 137.0 [M–H−150]^−^ and 109.1 [M–H−150–CO]^−^ for cyanidin (26, 27). Compounds 4-bromophenol (*m*/*z* 172.1) and 2,4-dibromophenol (*m*/*z* 250.7), respectively generated fragment ions at *m*/*z* 80.9 (peak 26) and *m*/*z* 81.4 and 78.5 (peak 27), which were consistent with previously reported mass spectral data (6, 7).

**Table 4:** Characterization of phenolic compounds from *Asparagopsis taxiformis* by UHPLC-MS.

**Peak No**.	**λ_max_ (nm)**	**Tentative compounds**	**ESI Model**	**Parents ions**	**Fragment ions**	**References**
1	275	Vanillin	+	152.99	138.0, 125.0, 93.0	(16)
2	280,310	Ethyl vanillin	–	164.95	136.2, 92.05	(17)
3	260,294	2-hydroxybenzoic acid	–	137.0	93.0	(18)
4	270,307	*p*-coumaric acid	–	163.1	119.0, 91.0	(19)
5	299,323	ferUlic acid	–	193.0	178.0, 149.0, 134.0	(19)
6	290,328	Rosmarinic acid	–	358.96	196.96, 161.0	(20)
7	278,306	*trans*-cinnamic acid	–	146.95	118.9, 77.0, 40.0	(18)
8	280	Catechin	–	289.07	244.9, 204.9, 137.1	(18, 21)
9	280	Epicatechin	–	289.07	244.9, 204.9, 137.1	(21)
10	270	Epigallocatechin	–	304.98	179.0, 124.98	(21)
11	270	Gallocatechin	–	304.98	179.0, 124.98	(21)
12	274	Epigallocatechin gallate	–	456.9	331.0, 169.0, 125.0	(21)
13	255,347	Quercetin	–	301.0	179.0, 151.0	(18–20)
14	254,352	Myricitrin	–	462.9	316.99	(11)
15	257,356	Quercitrin	–	447.0	301.0, 179.0, 151.0	(19)
16	256,354	Quercetin-3-O-α-L-arabinoside	–	432.9	300.9, 270.9	(22)
17	266,348	kaempferol-3-o-rutinoside	–	592.9	254.9, 284.8	(22)
18	283,327	Hesperidin	–	609.0	301.0	(19)
19	267,345	Diosmetin	+	301.0	257.0, 255.0, 153.0, 111.0	(24)
20	276	Baicalein	+	271.0	122.9	(22)
21	267,339	Apigenin	+	271.0	227.0, 151.0	(18)
22	270,335	Acacetin	+	285.0	153.0	(23)
23	292,330	Aromadendrin	–	286.8	258.97, 124.98	(25)
24	288	Leucocyanidin	+	306.9	126.99, 155.1	(26)
25	276,530	Cyanidin	+	286.9	109.1, 137.0	(27)
26	280	4-bromophenol	–	172.1	80.9	(6, 7)
27	280	2,4-dibromophenol	–	250.7	168.7, 81.4, 78.5	(6, 7)
28	–	Unknown	+	389.2	371.5	
29	–	Unknown	–	544.8	375.7	
30	–	Unknown	–	973.4	699.5, 617.2	
31	–	Unknown	–	265.0	220.9	

Although several studies have revealed the high TPC and total flavonoid content of *A. taxiformis* extract and the close relationship between them and the bioactivity (antioxidant, anti-inflammatory, and antiproliferative activities) (2, 5), the phenolic profile of *A. taxiformis* remains unknown. *Asparagopsis armata*, the sister species of *A. taxiformis*, has also shown strong antioxidant and antimicrobial activities together with high TPC and total flavonoid content (32, 33); however, studies focusing on its phenolic profile remain scarce. Notably, phenolic compounds have been reported in red, brown, and green algae (6, 30). Indeed, phenolic acids including ferulic, hydroxybenzoic, vanillic, salicylic, benzoic, gentisic, syringic, chlorogenic, caffeic, and protocatechuic acids were detected in red seaweeds including *Acanthophora specifera and Jania rubens* (34–36). Moreover, six flavonoids, including luteolin-5,7,3′,4′-tetramethyl ether, apigenin-7-O-glucoside, kaempferol-3-O-arabinoside, quercetin-3,7-dimethylether-4′-sulfate, catechin derivative, and dihydroxytrimethoxy flavone, were identified in the red seaweed *Alsidium corallinum* using LC-MS (37) Quercetin, vitexin-rhamnose, catechol, rutin, hesperidin, myricetin, and morin were detected in several red seaweeds, including *A. specifera*, using HPLC (35). Importantly, in brown algae, quercetin-O-hexoside, quercetin-O-glucoside, kaempferol-O-rutinoside, myricetin-O-rhamnoside, apigenin, baicalein, acacetin, diosmetin, and rosmarinic acid were identified by LC-MS (6, 8, 20, 22); notably, the first four compounds may be quercetin-3-O-α-l-arabinoside, quercitrin, kaempferol-3-O-rutinoside, and myricitrin, respectively, or their isomers. Flavan-3-ols, containing epigallocatechin, epicatechin, catechin, gallocatechin, epigallocatechin gallate, and catechin gallate, are abundant in brown seaweeds such as *Sargassum polycystum, Saccharina japonica, Sargassum fusiforme*, and *Eisenia bicyclis* (8, 38). Cyanidin, cinnamic acid, vanillin, *p*-coumaric acid, and other flavonoids (quercetin, apigenin, malvidin, kaempferol, lutein and myricetin) were detected in green seaweeds using LC-MS (39, 40). Furthermore, although the presence of leucocyanidin and aromadendrin (namely dihydrokaempferol) in seaweed has been scarcely reported, aglycone and glycoside forms of kaempferol and cyanidin were identified in both brown and green seaweeds (22, 39). Additionally, simple-structured bromophenols (e.g., 4-bromophenol and 2,4-dibromophenol) and relatively complex-structured bromophenols (e.g., nitrogen- and sulfur-containing bromophenols) were isolated and identified from diverse species of red seaweeds, such as *Rhodomela confervoides, Symphyocladia latiuscula*, and *Polyopes lancifolia* (6, 7).

#### Quantification of phenolics by UHPLC-MS

Following characterization, the phenolics were quantified using UHPLC-MS. Different extraction methods led to significant differences in the number and content of the individual phenolic compounds (Table 5). The numbers of individual phenolic compounds detected by the different UAE methods ranged from 11 to 26. DES-UAE detected 26 individual phenolic compounds, whereas only 11 phenolics were detected using Water-UAE. DES-UAE also gave the highest sum of the individual phenolic acid, flavonoid, and phenolic contents, which were respectively 6.74–153.11-, 1.17–4.62-, and 1.46–5.53-fold higher than the values attained with the other extraction methods (*p* < 0.05). DES-UAE and EtOH-UAE gave the highest sum of the individual bromophenol contents (*p* < 0.05). With respect to the ratio of the sum of individual flavonoid content to that of the individual phenolic content, Water-UAE and Acetone-UAE achieved values of 99.64 and 91.34%, respectively, whereas MeOH-UAE, EtOH-UAE, and DES-UAE achieved ratios of 81.82, 82.01, and 67.37%, respectively. These results revealed that flavonoids are the main components of *A. taxiformis*. This agrees with the results of Yoshie-Stark et al. (35), who reported that flavonoids are dominant in the phenolic profiles of several red seaweeds followed by phenolic acids.

**Table 5:** Quantification of individual phenolic compounds from *Asparagopsis taxiformis* by UHPLC-MS.

	**Content (**μ**g/g DW)**				
	**DES-UAE**	**Water-UAE**	**MeOH-UAE**	**EtOH-UAE**	**Acetone-UAE**
Vanillin	20.52 ± 0.54d	nd	15.14 ± 0.25c	5.51 ± 0.18b	3.21 ± 0.23a
Ethyl vanillin	0.02 ± 0.002a	nd	nd	0.05 ± 0.003b	nd
2-hydroxybenzoic acid	0.24 ± 0.01d	0.10 ± 0.004b	0.16 ± 0.01c	0.31 ± 0.02e	0.02 ± 0.001a
*p*-coumaric acid	5.28 ± 0.18e	0.16 ± 0.01b	0.36 ± 0.01d	0.07 ± 0.002a	0.26 ± 0.01c
Ferulic acid	74.34 ± 1.25a	nd	nd	nd	nd
Rosmarinic acid	2.10 ± 0.25d	0.45 ± 0.02c	0.47 ± 0.03c	0.02 ± 0.001a	0.05 ± 0.001b
*trans*-cinnamic acid	6.21 ± 0.24c	nd	nd	0.01 ± 0.001a	0.11 ± 0.01b
***Number of phenolic acids***	***7***	***3***	***4***	***6***	***5***
***Sum of individual phenolic acid content***	***108.71** **±2.35e***	***0.71** **±0.03a***	***16.13** **±0.26d***	***5.97** **±0.18c***	***3.65** **±0.23b***
Catechin	nd	nd	nd	0.03 ± 0.001a	0.12 ± 0.01b
Epicatechin	0.52 ± 0.03c	nd	nd	0.10 ± 0.003a	0.12 ± 0.01b
Epigallocatechin	0.84 ± 0.05d	nd	0.01 ± 0.001a	0.09 ± 0.002b	0.11 ± 0.01c
Gallocatechin	0.21 ± 0.01b	nd	nd	0.01 ± 0.001a	0.01 ± 0.001a
Epigallocatechin gallate	1.20 ± 0.05a	nd	nd	nd	nd
Quercetin	12.35 ± 0.27e	1.30 ± 0.13d	0.16 ± 0.01c	0.01 ± 0.001a	0.04 ± 0.002b
Myricitrin	0.54 ± 0.02e	0.24 ± 0.01d	0.05 ± 0.002b	0.02 ± 0.001a	0.08 ± 0.002c
Quercitrin	8.25 ± 0.44d	0.24 ± 0.01c	0.10 ± 0.01b	0.02 ± 0.001a	0.02 ± 0.001a
Quercetin-3-O-α-L-arabinoside	0.54 ± 0.03c	nd	nd	0.03 ± 0.001a	0.05 ± 0.002b
Kaempferol 3-O-rutinoside	0.21 ± 0.01c	nd	nd	0.01 ± 0.001a	0.05 ± 0.002b
Hesperidin	0.25 ± 0.01d	0.21 ± 0.01c	0.10 ± 0.01b	0.01 ± 0.001a	nd
Diosmetin	0.54 ± 0.02d	0.18 ± 0.01c	0.08 ± 0.002b	0.02 ± 0.001a	0.52 ± 0.04d
Baicalein	0.24 ± 0.01c	nd	0.01 ± 0.001a	0.07 ± 0.001b	0.01 ± 0.001a
Apigenin	0.55 ± 0.02b	nd	nd	nd	0.01 ± 0.001a
Acacetin	0.38 ± 0.01c	nd	0.01 ± 0.001a	0.04 ± 0.001b	nd
Aromadendrin	0.11 ± 0.001b	nd	nd	0.01 ± 0.001a	nd
Leucocyanidin	4.23 ± 0.22e	0.45 ± 0.02c	0.85 ± 0.03d	0.10 ± 0.01a	0.23 ± 0.01b
Cyanidin	335.89 ± 4.85d	255.24 ± 4.65b	78.06 ± 3.25a	301.35 ± 6.49c	313.25 ± 7.18c
***Number of flavonoids***	***17***	***7***	***10***	***16***	***14***
***Sum of individual flavonoid content***	***366.85** **±5.49d***	***257.86** **±4.79b***	***79.43** **±3.31a***	***301.92** **±6.50c***	***314.62** **±7.23c***
4-bromophenol	5.34 ± 0.25c	nd	0.10 ± 0.01a	0.44 ± 0.02b	nd
2,4-dibromophenol	55.67 ± 1.04d	0.23 ± 0.01a	1.42 ± 0.07b	59.80 ± 1.87e	26.18 ± 0.24c
***Number of bromophenols***	***2***	***1***	***2***	***2***	***1***
***Sum of individual bromophenol content***	***61.01** **±1.29d***	***0.23** **±0.01a***	***1.52** **±0.08b***	***60.24** **±1.89d***	***26.18** **±0.24c***
***Number of phenolics***	***26***	***11***	***16***	***24***	***20***
***Sum of individual phenolic content***	***536.57** **±9.13e***	***258.80** **±4.83b***	***97.08** **±3.65a***	***368.13** **±8.57d***	***344.45** **±7.61c***

Different letters in same row indicated significant differences (p < 0.05).

nd, not detected.

Among the individual phenolic compounds extracted by DES-UAE, cyanidin, ferulic acid, 2,4-dibromophenol, vanillin, quercetin, quercitrin, *trans*-cinnamic acid, 4-bromophenol, *p*-coumaric acid, and leucocyanidin accounted for 62.60, 13.85, 10.38, 3.82, 2.30, 1.54, 1.16, 1.00, 0.98, and 0.79% of the sum of the individual phenolic content, respectively. Cyanidin was the main phenolic component in the extracts obtained by Water-UAE, and accounted for 98.62% of the sum of the individual phenolic content. However, the main phenolic components of the extracts acquired by MeOH-UAE were cyanidin, vanillin, 2,4-dibromophenol, and leucocyanidin, which accounted for 80.41, 15.60, 1.46, and 0.88% of the sum of the individual phenolic content, respectively. Similarly, the main phenolic components acquired by EtOH-UAE and Acetone-UAE were cyanidin, 2,4-dibromophenol, and vanillin, which accounted for 81.86, 16.24, and 1.50% (EtOH-UAE) and 90.94, 7.60, and 0.93% (Acetone-UAE) of the sum of the individual phenolic content, respectively. The cyanidin content (78.06–335.89 μg/g DW) was the highest among all the individual phenolic compounds, with DES-UAE affording the highest cyanidin content (*p* < 0.05). Meanwhile, the 2,4-dibromophenol content varied within the range of 0.23–59.80 μg/g DW. EtOH-UAE afforded the highest content, which was 1.07-fold of that acquired by DES-UAE (*p* < 0.05). In addition, among all the tested extraction methods, DES-UAE afforded the highest contents of the other main phenolic components, including ferulic acid, vanillin, quercetin, quercitrin, *trans*-cinnamic acid, 4-bromophenol, *p*-coumaric acid, and leucocyanidin. These results confirm the high extraction efficiency of DES-UAE for extracting phenolics from *A. taxiformis*. They also show the significant differences in the phenolic profiles of the extracts obtained by the different extraction methods, which are in accordance with previously reported data (11, 13).

It is generally accepted that extraction methods contribute to the phenolic profiles and extraction yield (28, 30). Indeed, there are significant differences in the TPC, phenolic profiles, and antioxidant ability of *S. polycystum* extracts acquired by various solvents (11). Similarly, the phenolic profiles of *P. scandens* and *C. longa* are greatly affected by the extraction solvents 9, 13). In this study, we observed that the different extraction solvents (Bet-Lev, water, 70% ethanol, 70% methanol and 70% acetone), under the same UAE and extraction parameters, contributed to the variations in TPC, phenolic profiles, and antioxidant activities of *A. taxiformis*. These results imply that the extraction solvent has a significant effect on the phenolic extraction efficiency that is primarily derived from the difference in the solvent polarity (28, 30). Among the extraction solvents used in this study, Bet-Lev may have the most similar polarity to those of the phenolic compounds in *A. taxiformis*. This DES also has the advantage of strong hydrogen-bond basicity, which improves the intermolecularly interactive effects between the cellulose strands and Bet-Lev (10). Finally, the assisted extraction technology employed also contributed to the phenolic extraction efficiency. As a commonly used assisted extraction technology, UAE has been widely reported to enhance the extraction efficiency of phytochemicals, utilizing acoustic cavitation to interrupt the cell-wall structure (9, 12). Thus, we concluded that UAE using Bet-Lev as the extracting DES solvent is efficient in phenolic extraction from *A. taxiformis*.

## Conclusions

This study showed that UAE processes using different DESs afforded significantly different TPC, wherein UAE using Bet-Lev (1:2) as the extraction solvent afforded the highest TPC. The optimal extraction conditions obtained from the single-factor experiment and subsequent response surface methodology were as follows: water content of DES of 46.48%, ultrasonic temperature of 52.41°C, and liquid-to-solid ratio of 26.99 ml/g. Compared to the traditional solvent-based UAE, DES-UAE afforded the highest TPC and antioxidant ability and detected the most number of individual phenolic compounds and the highest sum of their contents. DES-UAE for phenolics from *A. taxiformis* is an efficient and environment-friendly method for the preparation of extracts rich in natural antioxidants, which may replace the synthetic antioxdants and widely be used in the fields of food processing, animal husbandry, and aquaculture.

## Data availability statement

The original contributions presented in the study are included in the article/supplementary material, further inquiries can be directed to the corresponding author.

## Author contributions

HG: methodology, project administration, and writing—original draft. YW: resources, investigation, and project administration. ZG: validation and writing—review and editing. YL: data curation and software. QW: formal analysis and funding acquisition. JX: conceptualization, funding acquisition, supervision, and writing—review and editing. All authors contributed to the article and approved the submitted version.

## Funding

This study was supported by the National Natural Science Foundation of China (31960483), the Natural Science Foundation of Hainan Province of China (320RC510), the Key Research & Development project of Hainan Province of China (ZDYF2021XDNY197), and the State Key Laboratory of Marine Resource Utilization in South China Sea (Hainan University) (No. MRUKF2021002).

## Conflict of interest

The authors declare that the research was conducted in the absence of any commercial or financial relationships that could be construed as a potential conflict of interest.

## Publisher's note

All claims expressed in this article are solely those of the authors and do not necessarily represent those of their affiliated organizations, or those of the publisher, the editors and the reviewers. Any product that may be evaluated in this article, or claim that may be made by its manufacturer, is not guaranteed or endorsed by the publisher.

## Abbreviations

*A. taxiformis*: *Asparagopsis taxiformis*
UAE: ultrasound-assisted extraction
DES: deep eutectic solvent
Bet-lev: DES synthesized with betaine and levulinic acid
TPC: total phenolic content
GAE: gallic acid equivalent
DES-UAE: ultrasound-assisted extraction with deep eutectic solvents
DW: dry weight
UHPLC-MS: ultra-high-performance liquid chromatography-mass spectrometry
Water-UAE: ultrasound-assisted extraction with water
MeOH-UAE: ultrasound-assisted extraction with 70% methanol
EtOH-UAE: ultrasound-assisted extraction with 70% ethanol
Acetone-UAE: ultrasound-assisted extraction with 70% acetone
FRAP: ferric-reducing antioxidant power
ABTS, 2: 2′-azino-bis(3-ethylbenzthiazoline)-6-sulfonic acid
Fe(II)E: FeSO_4_ equivalent
TE: Trolox equivalent.
